# GenSeizer: a Multiplex PCR-Based Targeted Gene Sequencing Platform for Rapid and Accurate Identification of Major *Mycobacterium* Species

**DOI:** 10.1128/JCM.00584-20

**Published:** 2021-01-21

**Authors:** Bing Li, Liyun Xu, Qi Guo, Jianhui Chen, Yanan Zhang, Wenpan Huang, Zhemin Zhang, Lizhong Han, Xiaogang Xu, Haiqing Chu

**Affiliations:** a Department of Respiratory Medicine, Shanghai Pulmonary Hospital, Tongji University School of Medicine, Shanghai, China; b Tongji University School of Medicine, Shanghai, China; c Shanghai Morgene Biotechnology Co., Ltd., Shanghai, China; d Department of Clinical Microbiology, Ruijin Hospital, Shanghai Jiao Tong University School of Medicine, Shanghai, China; e Institute of Antibiotics, Huashan Hospital, Fudan University, Shanghai, China; f Shanghai Key Laboratory of Tuberculosis, Shanghai Pulmonary Hospital, Tongji University School of Medicine, Shanghai, China; Carter BloodCare and Baylor University Medical Center

**Keywords:** *Mycobacterium tuberculosis*, nontuberculous mycobacteria, multiplex PCR, targeted gene sequencing

## Abstract

Mycobacterium tuberculosis and nontuberculous mycobacterium (NTM) infections often exhibit similar clinical symptoms. Timely and effective treatment relies on the rapid and accurate identification of species and resistance genotypes. In this study, a new platform (GenSeizer), which combines bioinformatics analysis of a large data set and multiplex PCR-based targeted gene sequencing, was developed to identify 10 major *Mycobacterium* species that cause pulmonary, as well as extrapulmonary, human diseases. The simultaneous detection of certain *erm*(41) and *rrl* resistance genotypes in M. abscessus was also feasible. This platform was specific and sensitive and exhibited no cross-reactivity among reference strains and a detection limit of 5 DNA copies or 50 CFU *Mycobacterium*/ml. In a blind comparison, GenSeizer and multigene sequencing showed 100% agreement in the ability to identify 88 clinical *Mycobacterium* isolates. The resistance genotypes, confirmed by whole-genome sequencing of 30 M. abscessus strains, were also correctly identified by GenSeizer 100% of the time. These results indicate that GenSeizer is an efficient, reliable platform for detecting major pathogenic *Mycobacterium* species.

## Abstract (precis)

Mycobacterium tuberculosis and nontuberculous mycobacterium (NTM) infections often exhibit similar clinical symptoms. Timely and effective treatment relies on the rapid and accurate identification of species and resistance genotypes.

## INTRODUCTION

Mycobacterium tuberculosis and nontuberculous mycobacterium (NTM) infections seriously endanger human health. According to the *Global Tuberculosis Report 2019*, M. tuberculosis infections remain one of the top 10 causes of death worldwide, with incidence and death rates of 130/100,000 and 16/100,000 people, respectively (1). In addition, a number of studies report a dramatic increase in NTM infections (newly described as a “neglected global threat”), which can cause refractory chronic lung disease with high morbidity and mortality rates (2). M. tuberculosis and NTM have similar microbiological properties and can cause infections that exhibit similar clinical symptoms. Therapies for these two infections, however, are different (3). Moreover, treatment options for different NTM species can be totally different (4, 5). Furthermore, effective drug treatment can vary sharply depending upon the subspecies and/or resistance genotype (4, 5). Therefore, the rapid and accurate identification of M. tuberculosis and NTM and the simultaneous detection of resistance genotypes are extremely important for the early diagnosis, treatment, and control of *Mycobacterium* infections.

Traditional etiological methods (e.g., acid-fast stained smears, biochemical tests, immunological tests, and culture) used to identify *Mycobacterium* species in microbiology laboratories are insensitive, time-consuming, and labor-intensive, which make them unsatisfactory in terms of the timely guidance of clinical treatment (6). A variety of molecular techniques have been developed to identify M. tuberculosis and NTM; however, these exhibit limitations in sensitivity, specificity, and/or efficiency (7–9). In contrast, technologies such as microarray and next-generation sequencing that rapidly analyze thousands of genes simultaneously can identify and genotype the resistance of the organism but are not cost-effective (10, 11). As such, a need remains to design and optimize specific genetic markers that identify and distinguish closely related *Mycobacterium* species.

Here, 10 species-specific sequences were screened, and an identification model was constructed by bioinformatics analyses of a large data set (whole-genome sequences of 8,095 strains belonging to 10 *Mycobacterium* species). Based upon this model, we partnered with Morgene Biotech (Shanghai, China) and developed a new multiplex PCR-based targeted gene sequencing platform, called GenSeizer, to identify 10 major *Mycobacterium* species implicated in human disease (M. tuberculosis, M. abscessus subsp. *abscessus*, M. abscessus subsp. *massiliense*, M. avium, M. intracellulare, M. kansasii, M. xenopi, and M. scrofulaceum among pulmonary pathogens as well as M. fortuitum and Mycobacterium marinum among extrapulmonary pathogens). The simultaneous detection of certain antibiotic resistance genotypes is also feasible. This platform has the advantage of simple operation, high identification efficiency, low cost, high throughput, and scalability. It will provide a rapid, reliable, and cost-effective test for laboratory diagnostics used in clinical settings.

## MATERIALS AND METHODS

### Bacteria.

Clinical isolates (M. tuberculosis, M. abscessus subsp. *abscessus*, M. abscessus subsp. *massiliense*, M. avium, M. intracellulare, M. kansasii, M. fortuitum, M. gordonae, M. smegmatis, M. scrofulaceum, and M. marinum) were obtained from Shanghai Pulmonary Hospital, Shanghai, China, and confirmed by 16S rRNA, *rpoB*, and *hsp65* or whole-genome sequencing (12). Ten reference strains, M. tuberculosis H37Rv (ATCC 27294), M. abscessus subsp. *abscessus* (ATCC 19977), M. abscessus subsp. *massiliense* (CIP108297), M. avium (ATCC 25291), M. intracellulare (ATCC 13950), M. kansasii (ATCC 12478), M. xenopi (ATCC 19250), M. fortuitum (ATCC 6841), M. scrofulaceum (ATCC 19981), and M. marinum (ATCC 927), were purchased from the American Type Culture Collection (ATCC) (Manassas, VA, USA). All strains were cultured in Middlebrook 7H9 liquid medium and incubated at 37°C. Streptococcus pneumoniae, Haemophilus influenzae, Enterococcus faecalis, Escherichia coli, Pseudomonas aeruginosa, Acinetobacter baumannii, Staphylococcus aureus, Klebsiella pneumoniae, and Nocardia asteroides were obtained from Shanghai Pulmonary Hospital, Shanghai, China, and cultured on blood agar plates.

### Species-specific gene sequence selection and identification model.

The entire genomes of 8,095 strains belonging to the 10 targeted *Mycobacterium* species were downloaded from NCBI GenBank (Bethesda, MD). To construct the identification model, 288 of these strains were selected at random (10-fold cross-validation was used to correct for unbalanced sampling) from the 10 targeted species (detailed data are shown in Table S1 in the supplemental material). For modeling, unified gene annotation of the genomes was performed using Prokka (rapid prokaryotic genome annotation v1.14.0) (13). Each strain was then given a standard gff (Generic Feature Format version 3) annotation file. Based on the information in these files, homology between genes was established using Roary2 (http://sanger-pathogens.github.io/Roary/) (14). Cluster analysis based upon homology was performed by Pearson correlation coefficient analysis; genes with a Pearson correlation coefficient equal to 1 were clustered. BLAST (v2.9.0+) was used to align representative sequences extracted from each genomic cluster of the 288 strains (the threshold was set to an identity of >85%, an E value of <1e−06, and coverage of >90%) in order to determine the presence or absence of a strain in the clusters, and the species-specific sequences were selected. Subsequently, a CART3 (classification and regression tree, rpart package in R language) decision tree was conducted to establish the identification model (15). Finally, the utility of the model was confirmed after the accuracy was verified for all 8,095 strains. NTM strains available in public databases but not included in the model (i.e., M. gordonae, M. smegmatis, M. chelonae, M. xenopi, M. simiae, M. neoaurum, M. gilvum, M. shimoidei, M. nonchromogenicum, M. kumamotonense, M. colombiense, M. triplex, M. phlei, M. gastri, M. vaccae, M. diernhoferi, and M. setense) were also included to confirm the specificity of the species-specific sequences after modeling.

### Detection of resistance genotypes.

The e*rm*(41) and *rrl* genes, which affect the macrolide sensitivity of M. abscessus, were added to the platform for resistance genotype detection. The amplified and sequenced DNA fragments of M. abscessus were aligned with the *erm*(41) (positions 2345955 to 2346476 of GenBank accession no. NC_010397.1) and *rrl* (positions 1462398 to 1463901 of GenBank accession no. NC_010397.1) reference sequences automatically, and the macrolide resistance genotype was then determined. The resistance genotypes include full-length *erm*(41) with T28 (without 64- and 65-nucleotide deletions or the deletion of nucleotides 159 to 432), *rrl* 2270C/G, and *rrl* 2271C/G (16, 17), which most commonly confer macrolide resistance. According to 2017 British Thoracic Society guidelines, treatment of M. abscessus infections is based on macrolide resistance.

### DNA extraction.

The Magen HiPure bacterial DNA kit was used to extract DNA from cultured bacteria according to the manufacturer’s protocol (Magen Biotech, Guangzhou, China).

To extract DNA from mycobacteria in artificial sputum samples, artificial sputum composed primarily of methylcellulose and emulsified eggs (Baso Biological Co., Ltd., Zhuhai, China) was divided into bottles, autoclaved, and stored at 4°C for 1 month. The initial concentration of five reference strains, i.e., M. tuberculosis H37Rv (ATCC 27294), M. abscessus subsp. *abscessus* (ATCC 19977), M. abscessus subsp. *massiliense* (CIP108297), M. avium (ATCC 25291), and M. intracellulare (ATCC 13950), was adjusted to an optical density at 600 nm (OD_600_) of 0.5 (about 10^8^ CFU/ml). One milliliter of the suspension was serially diluted 1:10, added to an equal volume of artificial sputum, and mixed evenly. Artificial sputum samples were treated with 3% NaOH and then neutralized with phosphate buffer and centrifuged. The pellets were collected, and the DNA was extracted by a rapid boiling method (18).

### GenSeizer assay.

The multiplex PCR-based targeted gene sequencing technology, called GenSeizer, was provided by Morgene Biotech Co., Ltd., Shanghai. The workflow is described briefly below.

### (i) Panel design and preparation.

*(a) Primer design and synthesis*. DNA sequences used in the identification model were selected as targeted fragments. Primer design and synthesis were conducted at Morgene Biotech and Sangon Biotech (Shanghai, China), respectively. All primers had relatively consistent melting temperature (*T_m_*) values. The formation of dimers or hairpin structures between primers or within the primer itself was avoided. Primers also included a linker sequence as a tag at the 5′ end. The length of all amplicons was relatively consistent. A recombinant plasmid (pUC57) containing full-length amplicon sequences was used to validate the primers and primer panels (Fig. S1).

*(b) Primer screening in a singleplex PCR system*. A singleplex PCR system and artificially synthesized recombinant plasmids containing the targeted sequences were used. The reaction mixture consisted of 10 μl 3× T enzyme mix (Morgene Biotech), 1 μl specific primer-F (10 μM), 1 μl specific primer-R (10 μM), 1 μl plasmid (1 ng/μl), and 17 μl nuclease-free water. The following PCR profile was used: denaturation at 95°C for 3 min; 30 cycles of denaturation at 95°C for 30 s, annealing at 60°C for 30 s, and extension at 72°C for 30 s; and a final extension step at 72°C for 5 min. The PCR products were identified by agarose gel electrophoresis.

*(c) Panel validation*. The initial panel consisted of all primers mixed in equal proportions (100 μM). Plasmid mixtures containing the same copy numbers of the targeted gene sequences were used for the amplification efficiency test. The proportions of the primers that were not uniform were adjusted until the following requirements were met: (i) the sequencing reads of all (100%) of the amplicons attained more than 10% of the average reads (average reads = total number of reads in the library/number of total primer pairs in the panel; for example, if the panel contains 20 primers pairs and the total number of library reads is 10,000, then the average number of reads is 10,000/20 = 500), (ii) the sequencing reads of 98% of the amplicons attained more than 20% of the average reads, and (iii) the sequencing reads of 90% of the amplicons attained more than 50% of the average reads. Primer panels that met the requirements described above were considered competent.

### (ii) Multiplex PCR.

*(a) Amplification and enrichment of targeted gene sequences*. The 30-μl reaction volume contained 10 μl 3× T enzyme mix, 8 μl panel mix, 2 μl bacterial DNA, and 10 μl nuclease-free water. The following PCR profile was used: denaturation at 95°C for 3 min; 25 cycles of denaturation at 95°C for 20 s, annealing for 20 s, and extension at 60°C for 4.5 min; and a final extension step at 72°C for 5 min. PCR products were purified after amplification using DNA purification magnetic beads (carboxyl-modified polymer magnetic microspheres) (Morgene Biotech). Briefly, 0.5× magnetic beads were first used to remove large DNA fragments, and 0.7× magnetic beads were then used to recover the targeted DNA fragments. The magnetic beads were washed with BW11 washing solution (Morgene Biotech) and 80% ethanol, and the purified PCR product bound to the magnetic beads was dried fully.

*(b) Sequencing adapter assembly*. Sequencing adapters for the Illumina platform were added to the purified DNA. The reaction mixture consisted of 10 μl 3× T enzyme mix, 1 μl 2F-barcode (10 μM), 1 μl 2R-barcode (10 μM), and 18 μl nuclease-free water. The mixture was added to the purified DNA product. The cycling conditions were as follows: denaturation at 95°C for 3 min; 8 cycles of denaturation at 95°C for 15 s, annealing at 58°C for 15 s, and extension at 72°C for 1 min; and a final extension step at 72°C for 5 min. The constructed DNA library was purified using DNA purification magnetic beads (0.9×); quality analysis was performed with an Agilent 2100 bioanalyzer (Agilent Technologies, Inc., Santa Clara, CA, USA). The DNA concentration was determined using a Qubit fluorometer (Thermo Fisher Scientific, MA, USA).

### (iii) Targeted gene sequencing.

Sequencing was conducted on a MiSeq system (Illumina, Inc., San Diego, CA, USA) with MiSeq reagent kit v2 (300 cycles). FastQ files were generated with MiSeq Reporter software.

### (iv) Offline data analysis.

Offline data generated by the MiSeq system were identified and counted through the adapter; reads with a double-end length of >60 bp were retained. Subsequently, the data were filtered; data with a Q30 of >50% of the reads were retained as high-quality data. Reads with <60 bp on either end, single-end recognition of the primer, or nonspecific primer binding were rechecked and deleted as determined from the high-quality data. Clean read pairs for identification and sequence alignment were obtained as a result.

### Evaluation of specificity and sensitivity (limits of detection).

Ten mycobacterial reference species and nine nonmycobacterial bacterial species (E. coli, P. aeruginosa, A. baumannii, S. aureus, K. pneumoniae, S. pneumoniae, H. influenzae, E. faecalis, and N. asteroides) were used to assess the specificity of GenSeizer. The limit of detection (LOD) was evaluated in terms of both DNA and CFU of five reference strains: M. tuberculosis H37Rv (ATCC 27294), M. abscessus subsp. *abscessus* (ATCC 19977), M. abscessus subsp. *massiliense* (CIP108297), M. avium (ATCC 25291), and M. intracellulare (ATCC 13950). DNA extracted from these cultured strains was serially diluted ranging from 100,000 to 5 copies. Mycobacteria in artificial sputum samples were diluted from 50 to 1 × 10^6^ CFU/ml, and the DNA was extracted. GenSeizer analysis was conducted to ascertain specific DNA amplification and the LOD. Specificity and sensitivity assays were repeated 3 times.

### Application of the GenSeizer platform to clinical *Mycobacterium* isolates.

To determine the feasibility of using the GenSeizer platform to identify the major *Mycobacterium* species, a total of 88 clinical isolates (30 M. tuberculosis, 20 M. abscessus subsp. *abscessus*, 10 M. abscessus subsp. *massiliense*, 10 M. avium [subsp. *hominissuis*], 5 M. intracellulare, 3 M. kansasii, 1 M. xenopi, 3 M. fortuitum, 3 M. scrofulaceum, and 3 M. marinum) were examined in a blind study. The entire genomes of the 20 M. abscessus subsp. *abscessus* and 10 M. abscessus subsp. *massiliense* isolates were sequenced previously by us (DDBJ/ENA/GenBank BioProject accession no. PRJNA398137); their resistance genotypes were confirmed (19). As such, these isolates were used to assess the ability of GenSeizer to evaluate genotype resistance.

## RESULTS

### Identification of targeted gene sequences.

Among the 288 strains used for modeling, 111,729 homologous gene clusters that converged into 12,893 classes were identified (data not shown). Applying the CART decision tree algorithm, 10 sequences with the best overall sensitivity and specificity were identified and used (Table 1; see also Table S1 in the supplemental material). The identification model is shown in Table 2. The accuracy was then tested for all 8,095 strains downloaded from NCBI GenBank; the model showed a high degree of accuracy ranging from 97.6% to 100%. Further analysis indicated that these sequences did not misidentify NTM species that were not included in the model (data not shown).

**TABLE 1:** Species-specific sequences of each reference strain, genome locations, and brief gene descriptions

Sequence no.	Mycobacterium	GenBank accession no., sequence positions	Gene description
1	M. tuberculosis	NC_000962.3, c13176–13024	Membrane protein
2	M. abscessus subsp. *abscessus*	NC_010397.1, 2818383–2818925	Cytochrome *c* oxidase subunit III
3	M. abscessus subsp. *massiliense*	AP014547.1, c469656–469192	PadR family transcriptional regulator
4	M. avium	NZ_AP012555.1, 1840894–1841223	STAS domain-containing protein
5	M. intracellulare	NC_016946.1, 2010043–2011506	Acyl-CoA ligase
6	M. kansasii	NC_022663.1, c344353–344087	Hypothetical protein
7	M. xenopi	NZ_AP022314.1, 4571470–4571946	Glycohydrolase toxin TNT-related protein
8	M. fortuitum	NZ_CP011269.1, 1731500–1732405	LysR family transcriptional regulator
9	M. scrofulaceum	NZ_LZJW01000054.1, 4417–4734	Fe-2S iron-sulfur cluster binding domain-containing protein
10	M. marinum	NZ_HG917972.2, c12988–12227	MerR family transcriptional regulator

**TABLE 2:** Identification model: sequences expressed by different species

*Mycobacterium* species	Primer sequence^*a*^ (forward and reverse)	Targeted gene sequence no.^*b*^									
		1	2	3	4	5	6	7	8	9	10
M. tuberculosis	5′-CTACGTCGGCTCGTCGCTC-3′	●	×	×	×	×	×	×	×	×	×
	5′-GCCAAAGGTGAGCGGACTTG-3′										

M. abscessus subsp. *abscessus*	5′-CTTTGAATACGGTCGCCATCTGAC-3′	×	●	×	×	×	×	×	×	×	×
	5′-GATACCTTCCAGTAGAGCTACGCC-3′										

M. abscessus subsp. *massiliense*	5′-GAGAAGACACTGGCCCGATTCA-3′	×	×	●	×	×	×	×	×	×	×
	5′-TGGTTCCTTCCTTACGGTCTTGAG-3′										

M. avium	5′-GAAATCGACCTGAGCAACATCGAC-3′	×	×	×	●	×	×	×	×	×	×
	5′-TCAAACCGCTGACCCTGAAGAC-3′										

M. intracellulare	5′-CAACGTTTCTCGACTCATCACCTG-3′	×	×	×	×	●	×	×	×	×	×
	5′-GACGCATTTTCCAAGCCAGGTTTC-3′										

M. kansasii	5′-ATGCCTGGTGTATCTGCAGCAAAT-3′	×	×	×	×	×	●	×	×	×	×
	5′-TTTCCTGAGGGTGTTGATCGTGTT-3′										

M. xenopi	5′-CCAAATCCTCTTCAGCTCTACCGA-3′	×	×	×	×	×	×	●	×	×	×
	5′-CTTATAACCCTGGTCGGCTTTCGA-3′										

M. fortuitum	5′-CAGCTGATCACCTTTTCGTCGAC-3′	×	×	×	×	×	×	×	●	×	×
	5′-GAATCAGAGCCACACCCAATCCC-3′										

M. scrofulaceum	5′-ATGGCAGATGTCGAAGAACAAGGC-3′	×	×	×	×	×	×	×	×	●	×
	5′-CAATGTCCTTCACGACACGAGTG-3′										

M. marinum	5′-ACTGGAAGTTGATCGTCGAGAACT-3′	×	×	×	×	×	×	×	×	×	●
	5′-GTTGATGAACACCGTCGGTTTGAC-3′										

a Primers used in the multiplex PCR assay to target a specific *Mycobacterium* species.

b Distribution of the 10 targeted gene sequences among the species indicated. ●, present; ×, absent.

### The GenSeizer platform.

**FIG 1:**
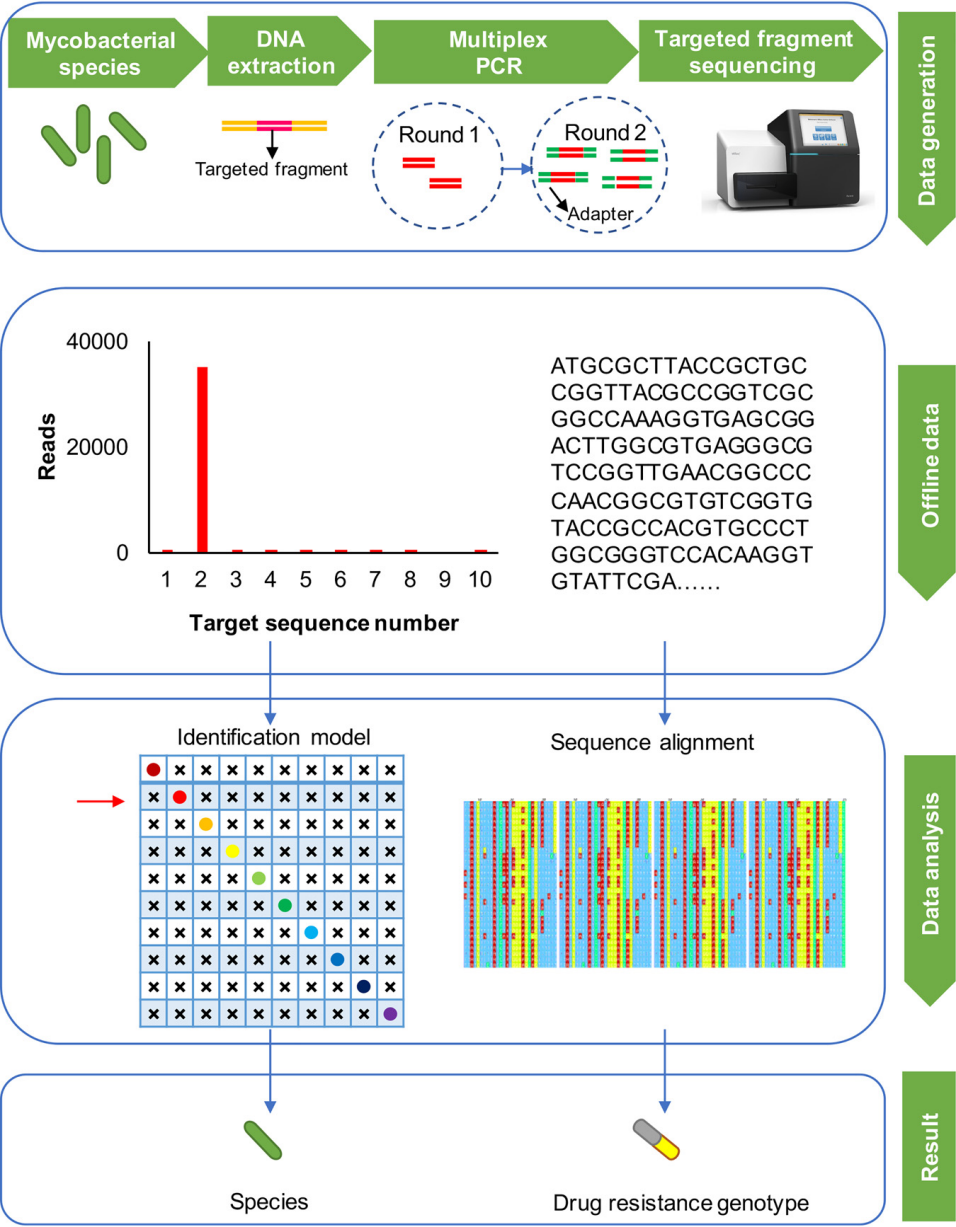
Schematic of the GenSeizer platform. First, the DNA was extracted. Targeted DNA fragments were then amplified and connected with sequencing adapters by two-step multiplex PCR. Targeted gene sequencing was performed, and both reads and the amplified gene sequences were obtained. Offline data were automatically converted to species identifications and resistance genotypes by a program that was specifically designed based upon the identification model and sequence alignment.

A schematic overview of the GenSeizer platform is shown in Fig. 1. Targeted DNA fragments were selected from our identification model. The targeted fragments were amplified by multiplex PCR (primers are shown in Table 2), the enriched targeted fragments were connected with sequencing adapters, and the targeted genes were then sequenced. The offline data generated by the sequencing system contain both the amplicon counts and sequences (reads) of the amplified fragments. These data can be used to identify the species and determine the resistance genotype of an isolate by applying a program specifically designed based upon the identification model and sequence alignment.

### Evaluation of the GenSeizer platform.

**FIG 2:**
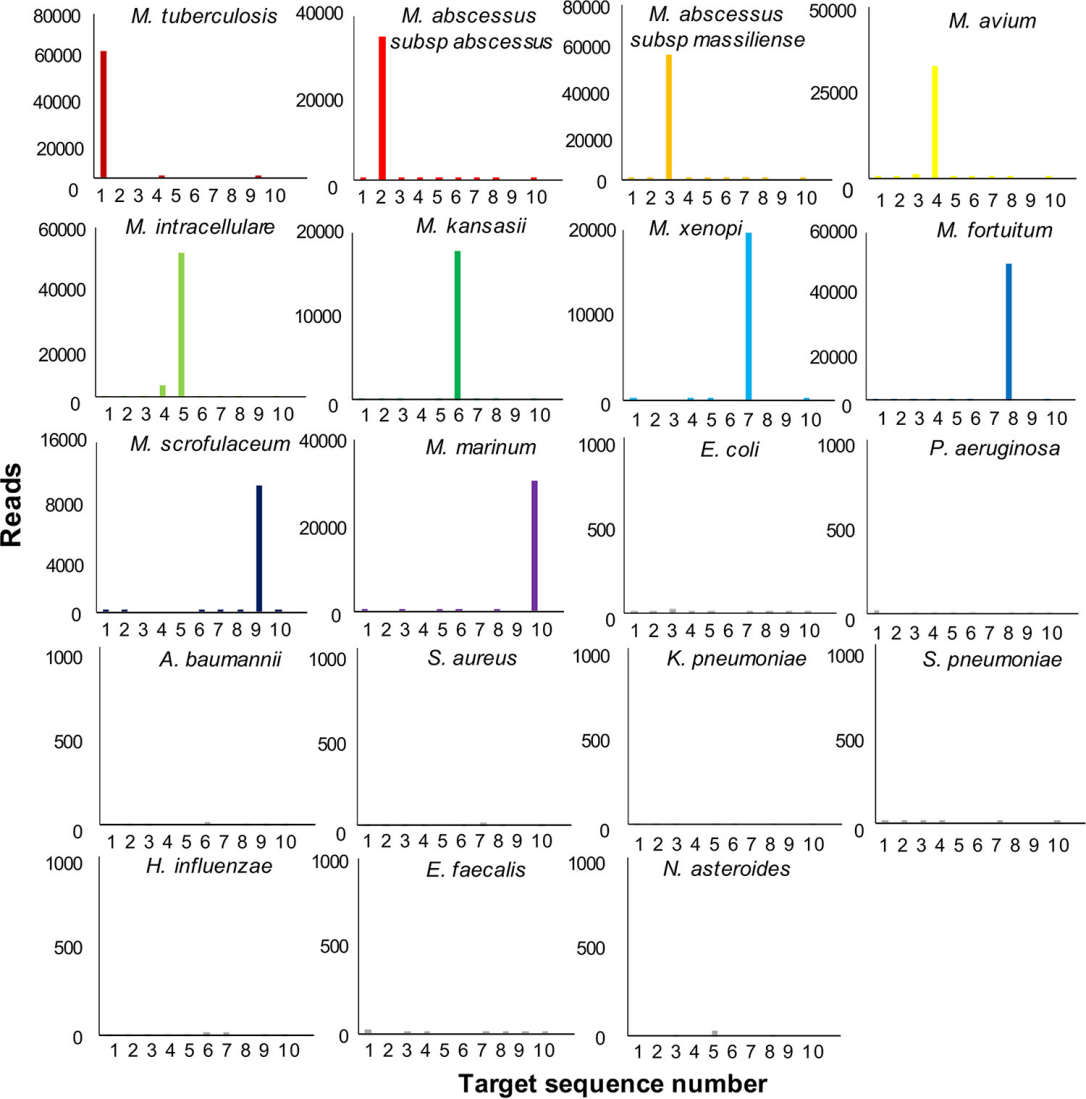
GenSeizer specificity. Ten *Mycobacterium* reference strains and nine nonmycobacterial strains isolated from the lower respiratory tract were used to evaluate GenSeizer specificity and the targeted gene sequence. Each assay was repeated three times.

**FIG 3:**
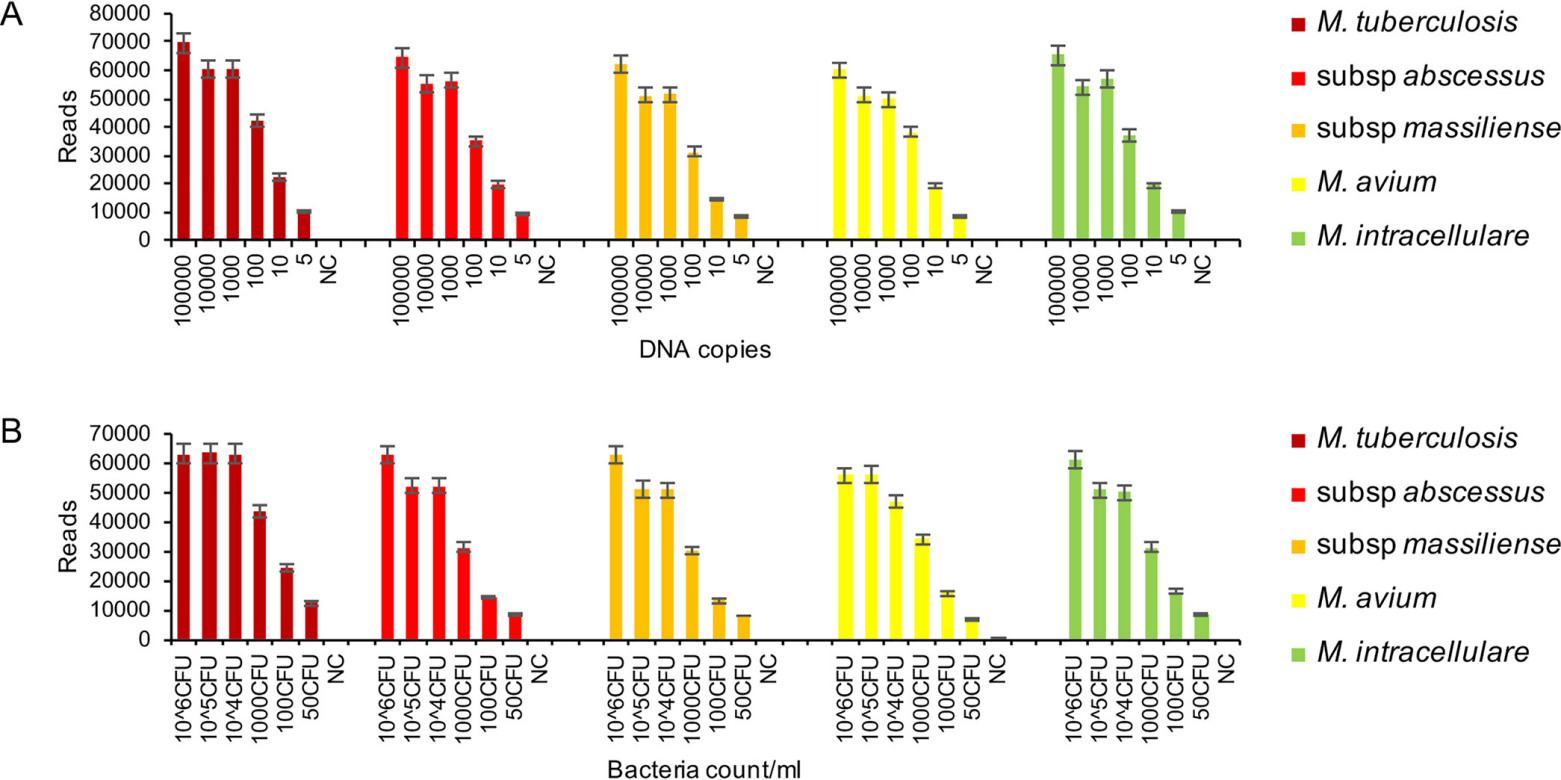
Limits of detection. The LOD was evaluated using the five major *Mycobacterium* species indicated; E. coli served as the negative control (NC). (A) The LOD was evaluated with DNA extracted from pure, cultured reference strains. (B) The LOD was evaluated with DNA derived from the indicated number of organisms in artificial sputum. Each assay was repeated three times.

The specificity of the GenSeizer platform was evaluated using 10 *Mycobacterium* reference strains. Nine nonmycobacterial species isolated from the lower respiratory tract were used as controls. The results shown in Fig. 2 indicate that the GenSeizer platform can specifically identify *Mycobacterium* species without cross-reaction. The amplicon counts for detecting specific *Mycobacterium* sequences were much higher than those for the control organisms (14,917 to 61,896 versus 0 to 33 reads). Additionally, GenSeizer was very sensitive in detecting different *Mycobacterium* species exhibiting similar LODs of 5 DNA copies (Fig. 3A) or 50 CFU/ml (Fig. 3B). The results obtained for clinical isolates were comparable (data not shown).

### Application of the GenSeizer platform.

GenSeizer was used to identify clinical *Mycobacterium* isolates. A cutoff value for each sequence was established based upon a nontargeted mycobacterial species. By these criteria, GenSeizer correctly identified the species of all clinical isolates, showing 100% agreement with previous 16S rRNA, *rpoB*, and *hsp65* sequencing results (data not shown). The *erm*(41) and *rrl* antibiotic resistance genotypes of the 30 M. abscessus isolates (previously sequenced entirely) were also identified correctly 100% of the time by GenSeizer (Table 3).

**TABLE 3:** Comparison of *erm*(41) and *rrl* resistance genotypes detected by GenSeizer versus whole-genome sequencing^*a*^

Platform	No. of isolates				
	Full-length *erm*(41) T28	*rrl* 2270C	*rrl* 2270G	*rrl* 2271C	*rrl* 2271G
GenSeizer	18	2	0	0	1
WGS	18	2	0	0	1

a Data are the numbers of isolates among a total of 30 M. abscessus isolates that exhibit the resistance genotypes indicated. WGS, whole-genome sequencing.

## DISCUSSION

M. tuberculosis infection is recognized among infectious diseases as the leading cause of death; the mortality rate is higher worldwide than for HIV and malaria (1). The incidence and prevalence of NTM disease are increasing dramatically. In some developed countries, NTM infections even surpass the global incidence of new tuberculosis infections (2). M. tuberculosis and some NTM infections are clinically indistinguishable. *Mycobacterium* species differ dramatically, however, in terms of treatment outcomes and antibiotic susceptibilities (4, 5). Moreover, NTM consist of more than 200 species; reportedly, ∼5% are pathogenic for humans, and more than 10 NTM are particularly important to immunosuppressed populations. Optimal treatment strategies rely on early species identification and determination of drug susceptibility (5, 20). Consequently, developing a method for rapidly and accurately detecting major *Mycobacterium* species, as well as predicting antibiotic sensitivity based upon genotype, is extremely relevant clinically.

The current study describes the development of a new platform (GenSeizer) that combined bioinformatics analysis of a large data set and multiplex PCR-based targeted gene sequencing. Importantly, 10 *Mycobacterium* species-specific gene sequences were identified by analysis of a large public database, ensuring the greatest specificity possible. Analysis indicated that these sequences did not misidentify NTM species that were not included in the model, thus circumventing a major problem. Specific primers were designed based upon these sequences; upstream multiplex PCR enhances the amplification of the targeted sequence, providing sufficient sensitivity. The high-throughput sequencing downstream further evaluates both the counts and the sequence information of the amplicons. Sequenced amplicons as a detection signal can improve accuracy because nontargeted gene amplicons can be removed. The sequenced amplicons present the resistance genotypes simultaneously; detection specificity is also guaranteed. Since the amplified product does not contain nucleic acid of human or other microbial origins, the amount of data required for a single sample library is only 0.01 million to ∼0.03 million reads (metagenomic next generation sequencing usually requires at least 20 million reads for each sample library), which greatly improves the detection throughput and reduces the sequencing cost. The multiplex PCR technique makes it possible for this platform to place 1,000 primers in one reaction tube (21, 22). New targets can be added at any time as needed clinically by adding new primer pairs to the specific primer set; the cost is almost unchanged. Therefore, the platform exhibits good sensitivity, specificity, cost-effectiveness, and scalability.

In this regard, the GenSeizer platform showed a high degree of specificity comparable to those of current, multiple homologous gene sequencing approaches. Furthermore, the cross-reactivity was nil, and the accuracy of identifying 8,095 strains in the public database at the genome level was high (97.6 to 100%). A high rate of accuracy was similarly found for 10 reference strains (100%) and 88 clinical isolates (100%). High-throughput sequencing was a simple process (3-h protocol with 30 min of hands-on time), and the cost was low (∼$5 per sample). Moreover, the LOD of 5 DNA copies or 50 CFU bacteria/ml confirms the extreme sensitivity of this platform. Chae and colleagues constructed a platform similar to the one described here using new sequence-specific markers combined with a one-step multiplex PCR system that could identify M. tuberculosis and five NTM species, including M. abscessus subspecies (22). Their assay could also detect the especially widespread M. tuberculosis Beijing genotype, supporting the feasibility of our platform and approach.

The presence of macrolide resistance genotypes adversely affects the prognosis of M. abscessus-infected patients (23–25). The British Thoracic Society guidelines released in 2017 recommend that the treatment of pulmonary M. abscessus infection should differ depending upon the macrolide susceptibility of the infecting subspecies (5). Targeted therapy dependent upon the resistance genotype is a trend toward “precision treatment” (26). The treatment of M. abscessus infections based on the macrolide resistance genotype is emblematic of this trend. Importantly, the GenSeizer platform can simultaneously detect certain antibiotic resistance genotypes, i.e., *erm*(41) and *rrl*, in addition to 10 *Mycobacterium* species and subspecies. These resistance genotypes were detected in 30 clinical M. abscessus isolates with 100% accuracy. Notably, GenSeizer is of high throughput (over 1,000 primer pairs can be included in one tube). As such, the detection of resistance genes can be upgraded and expanded, thereby increasing the power and promise of the platform.

The present study has several limitations. First, the selected sequences did not reach 100% identity for all *Mycobacterium* strains in the public database; a few strains were not identified. The failure to identify these few strains was mainly due to the absence of complete sequences in the public NCBI GenBank database. Information regarding these strains is provided in Data Set S1 in the supplemental material. Second, the number of some NTM species used for model design and verification was too small due to the limited involvement of these species in clinical infections. Third, some clinically important species were not included since the corresponding reference or clinical strains were not available in our library. Finally, the fact that freshly isolated clinical specimens were not used to evaluate the GenSeizer platform is a matter of ongoing investigation. In this regard, a clinical trial led by us is currently in progress (ClinicalTrials.gov identifier NCT03224065). In conclusion, GenSeizer facilitates the rapid, cost-effective, sensitive, and specific identification of M. tuberculosis, major NTM species, and certain antibiotic resistance genotypes.

## Supplementary Material

Supplemental file 1

Supplemental file 2
