# Supplementary material for: GenSeizer: a Multiplex PCR-Based Targeted Gene Sequencing Platform for Rapid and Accurate Identification of Major Mycobacterium Species
Source: J Clin Microbiol. 2021 Jan 21;59(2):e00584-20. doi: 10.1128/JCM.00584-20 (PMC8111139; doi:10.1128/JCM.00584-20)

Supplementary Table S1. Species distribution of all 8,095 strains, and the 288 strains used for modeling

Mycobacterium species	Strains[a]	Strains used for modeling[b]
M. tuberculosis	6,533	80
M. abscessus subsp. *abscessus*	869	50
M. abscessus subsp. *massiliense*	360	23
M. avium	210	50
M. intracellulare	23	15
M. kansasii	42	30
M. fortuitum	24	18
M. gordonae	6	6
M. smegmatis	22	15
M. scrofulaceum	3	3
M. marinum	25	20

[a]Data are the total number of strains of the species indicated that were downloaded from NCBI GenBank.

[b]Data are the of number of strains of the species indicated that were selected at random and used to construct the identification model.

Supplementary Table S2. Ten targeted sequences used in the identification model

Sequence Number	Base sequence
1	ATGCGCTTACCGCTGCCGGTTACGCCGGTCGCGGCCAAAGGTGAGCGGACTTGGCGTGAGGGCGTCCGGTTGAACGGCCCCAACGGCGTGT<br>CGGTGTACCGCCACGTGCCCTGGCGGGTCCACAAGGTGTATTCGAGCGACGAGCCGACGTAG
2	GTGCCCGCAGAAGCAGAGCTGTGGATCTTCATCATGGGAGATCTCACCGTGTTCGGCGTCTTCTTCGCGGTCTGGGGATGGAGCTACGCCCG<br>CAACCCGGCCATGTTCGAGCTCGGCCGCGCGAGCATGTCGCAGTCGATCGGTCTGACCGAGACCCTCACCCTCATCAGCAGTTCTGCTGCGG<br>TGGTCACCGCGCTTACCCGTGCGCGGTGGAAACAATGGTCCCGGGCGTGCGCCTGGTACCTCACCGCAATCGGTTTCGGTGCGGTGTTCGTG<br>GTGTTGAAAGCCTTTGAATACGGTCGCCATCTGACCACCGGCCTGGATCCCCTCGCGGGTGAATTCTTCATGTATTACTTCGTTTTCACGGGA<br>ATTCACCTGCTGCACGTACTGATCGGACTCCTCGGACTGGGCGCTGCCGCGCGGTCGATGCGCCCTAGAGCCACACGTCGCTATGGCGTAGC<br>TCTACTGGAAGGTATCGGCGTGTACTGGCACATGGTGGATGTCTTATGGGTCGTGCTGTTCTCGTTGATCTACCTGATCTAG
3	ATGGCACGGTTCTTCCGGCATGGCGAGCTGCCACTGGTGCTTTTGGCACTGTTGGCCCAGCGGCCTATGCATGGATATGAGCTGATGTCCGAG<br>TTGTCGCGCCTTTTCGGCCCCGGCGGGTATCAGCCGTCCCCCGGAACGGTGTATCCGGCGGTTGATGCACTGGCGATGGAAGGTCTGCTGAT<br>CGGCCAGGCGCGCGAGGGTCGAACGGTCTACCGTGCCTCCGCCGAGGGCGAGCAGGCCCTGATCAGCCGCGCCGATGTGCTGGCGACCGTT<br>GAGTTACGCACCGGTGCGCGGTTCGGCCGCGGCGATTCGCTCGAGAAGACACTGGCCCGATTCACCGCTCGAGTCATGCCGTATGCGGGTCG<br>GGTGGAACCCGCAGCGGCAGAAGCAATCCTCGATCAGGCCGCCGCCCAGATCGCGGATCTTGTCCGGACTCAAGACCGTAAGGAAGGAAC<br>CAAGTGA

4	ATGGTCACCCCGCTGACGCTGGACACCGGCCGTGGTAGCGACGGCAACCCGGTGCTGGTGGCAGTGGGCGAAATCGACCTGAGCAACATCG<br>ACGCATTCCACCGGGCGCTGGCCACCGCCACCGCGGAGGTCACCGGGAGTGACGGCGCGGTGCTCGTCGACCTCAGCGCCGTGGAGTATGT<br>GGACAGCGCCGCCATCAATGCGTTGGCCGCGCACGCCGACCACATCGCGCTCGTCGCGCACCCGGTCCTGATGCCCGTCTTCAGGGTCAGC<br>GGTTTGACCGAGCTGACCACCGTCGAAGCCGCACCCCCGCCGCCGGCGCCTCGTTGA
5	GTGAACCTCGCAACGCTGCTGGACGGTCTACCCGACGTCGCGGTGCACACGGTCGGCGCCGAGGTATCCCGTGACGAGCTGTCGGCCAGTG<br>CCCAGCGACTCGGCAAGCTGCTCGCCGAGTCCGGGCTGGCGACGGGACAGGTCGTCGCCGCGATGCTGCCCAACGACGCCACCACCATCGC<br>CGCGCTGTTCGGCACGTGGCACGCCGGCGGCGTGTACACACCGCTAAACCCCAGGGCGGCCGATCCCGAGATCCTCGCCCAGCTGGAAACG<br>CTGCGCCCCGTTGCCATCATCACGACCCCCGCACTCGCACAACGTTTCTCGACTCATCACCTGCCCGTAATCACCGGCGAGGCGCTGTCGTG<br>GACCAGGGTCGCGTCGTCAGGCCACTCGGCCGGCGAGCCGTGCTACGACGACGACGTCGCGCTGCTGCAGTTCACTTCCGGCACCACGGG<br>ACCGCCGAAGCCGGTGCCGCTTCGTCACAGCACGGTGCTCGATCTGATCGACCGGCTGCTGGCCAAACTTCGCGGCACGAAACCTGGCTTG<br>GAAAATGCGTCGGCCCCACCGAAGAGGCCCCCCATGCCGAACCTGGTGCCCTTGTCGTTGTCGCTGTGGGCCGGCATCTACCAGGTGCTGTT<br>CGCGTTCCGGGCCGGTTCGGGCGTCGTGCTGATGGACCGGTTCTCACCGACGGATTTCGCCGCGCTGGTCCGGCGCCACCAGCTGCGATCCA<br>CGGTGCTGCCGCCCGCGGCGCTGACCATGGTTCTGCACGACGACTCGCTGGCCGACCTCTCGCCACTGAAGATCGTGCGGTCGATCACCGCT<br>CCCCTGTCGCCCGTGCAGGCGCGCCGGTTCCGGGACAAATTCGGGGTGATAGTGCTGAACTCCTACGGCCAGACCGAACTCGGGGGTGAGG<br>TGGTGGGCTGGTCGGCCGCCGACGCCCGGGAGTGGGGCGAGACCAAACTGGGTTCGGTCGGCAGGCCGCTGCCCGGCATCGACGTCACAA<br>TCGCCGACGACGAGGTGATGGTCCGCACGCCCACGACGGCCGCCCGGAAGATCGATCCAGCCTTCCTGGACCGGCTGACCGACGACGGCTG<br>GTTCCACACCGGCGACCTCGGGTGGTTCGACGACGACGGCTTTCTGTGGCTCGACGGCCGAGTATCGGACATGATCAATCGTGGTGGCCTCA

	AAGTGTTTCCCGGCACGGTAGAGGACGTCCTGCTGGCCGCCGATGGGGTGCGCGAAGCCGCCGTCGTCGGCGTTCCGGACGAGCGGCTCGG<br>GGAGGTGCCGTGGGCGTTCGTCGTCAGAGGCGATGAGTCGGTCTCCGAGGCCGGCCTCGTGTCGTGGTGTCGAGAGCGTCTGACGCCCTAC<br>CGGGTTCCGGTACGCATCGTGTTCGTCGAACAGCTGCCCCGCAACGATGTCGGCAAGGTGGTCAAGCGCGACCTCGCTGCGCTCGCGGACG<br>TGTGA
6	ATGACCGTCTACGACAACACGGTCCCCGCCGTCGACTGCGTTGATTTCGTCCGACTCGTCGACGACCTGGTGGACTCGGACCCGCAGGAGT<br>GGGGAGCGATCGTGGCCAAACACATCGACGAGTGCCCGCCATGCCTGGTGTATCTGCAGCAAATGCTGGACCTCAAGGTTCTGCTCAATCAC<br>GTATTCGATGGGGAAAAGCTCAGCGACGAGCACATCGCGGGGGTTATCAACACGATCAACACCCTCAGGAAAGGCCAAGAATGA
7	ATGGTAGACTTCTCTCGCTTATCGACAAATTGGTACCAATGGGCGCCCGGGATGAGCGGCGGCGATATATCTGTGTCGACCGACTGCGAAGAC<br>TGCCAAATCCTCTTCAGCTCTACCGATTATTCGGTGCATCTCCGCAATGATGGCCATTGGTGGGTCGTGGACATCGTCAACGACCGTGGGCAG<br>CGGCGGAGTGGCGCTGCCAAACTCTCAAGTTTCGAATTGACGGAAAAGTACCTTATCTGGGACTGGGCGACTACCGCGCGTTCAAGTCTGG<br>CATCTGGACCGTTAGGCACTGACCTAGCTAGGCAAGGATACGCGCCTGACGTTGAAGTTTCGAAAGCCGACCAGGGTTATAAGATATGTTTG<br>GGTAATGAATGCGCAATACTATCGGTCGTTAACGCTACAATTTTTAGTCATCTAATGTCGAAATCGGTGGATGAAATTGAGCATATGGTATACG<br>AAGGTCTCATGTAG
8	ATGGAGCTGCGACAGCTGGAGTACTTCGTGGCCGTCGCGGAAGAGGGCAGCTTCACCAAGGCGGCCGCGCGCATGCACATCGCTCAATCGG<br>GTGTGAGCGCTCAAATCCTGCTACTGGAGCGTGAACTCGGTCAGCGGCTCTTCGACAGGTCACGTTCTCCGGTGCGGCTCACCAAGGTCGG<br>TACCGCCTTGCTGACGCACGCGCATGCCGCGCTCGCCGCGGTGGCCGACGCCAGGCAGGTAGCAGACGAGTACGGCGCTGCCTTGCGCGGC<br>CACGTCAGCGTCGGCCTCGCGGCATCGTCGTCGCTCGCTTTCGATCTGGTCGACATGTTGGCGGAGTTTCACCGCGCACACCCCATGGTCGA

	GATCGCTCTGTTGGAGGCGAACACCGATGATCTCGTCGACGGGTTGCTCGACGGACGCCATGACATGGCGATCATCTCGCCGCCGGCGACGG<br>TGCCTGCTGATCTTCGCCTGCAGCTGGTGGCGGACGAGCAGATCGTCGCCGCGGTAAGTTCCGACCATCCGCTGTGCGGACGCTCGGGCGTG<br>ACACTCGAGGAGCTGAACGGCTGCCAGCTGATCACCTTTTCGTCGACGATCGGGACTCGCAGCACCATCGATGCCGCGTTCGCGGCCGCCG<br>GCATCCAACCTCATGTCGCCATCGAGGCCAGTGACCCGAATGTCCTCGCCGAATTGGCGGGCGGGGGATTGGGTGTGGCTCTGATTCCCGAG<br>CCGTATGCGCGTGCGCGTGATTCGAGGCTGCACGTCATGCAGATCGCGGACGTGGATCTGCGCGGCAGCTTGGCGTTGGTGTGGAACGACG<br>CCAGGAAACCGAGTACCAGTGCGGCCCGATTGGTCGACTACGCGCGACGTTCCTTGCGATCGGGTCAGGCGTTGTCGCCCTGA
9	ATGGCAGATGTCGAAGAACAAGGCGCTGCGCTGTGCACCGACGTCGAGGCAGCCACCGTCGACGTCGAGTTGGACGGTGCTCTCCATCGAC<br>TGCAATGGCGCCGCGATCGGACCCTGGTCGACACGATGCTTGACGCCGGGATCAATGTGCCGCACTCGTGTCGTGAAGGACATTGCGGCTCC<br>TGCGTGGCGACCCTGATTTCCGGGGAGGTGGAGATGGCCGGTGGAGATGTGCTGGGACCCGAAGACCGCGCCGACGGCTTGATCCTCGGCT<br>GTCAAGCCCGGCCAGTGACAGACAACATCCACATTGAGTTCTAG
10	ATGACCGCTGTCCGGCAACCCTCGGCCTTGCTGCCCACCCTGGGCGGGGCCTATTACACCAGTGCCGCCGTGTTTGCCGCCGAGCAACAGCA<br>CGTGTTCGAAAGCATGTGGTTTTGCGTCGTGCGCGCGGCCGACCTGAACGAGCCGGGGCAATTCAAGGCCGTACAGGTGGGACGCGAAAG<br>CGTGCTGCTGGTGCGGGGGCGGGATCGACGGCTGCGCGCCTTTCTCAACATCTGCCGGCATCGAGGTGCGCTGCTGTGCACCGAGCCCGAG<br>GGACAGGTGCGCCGGAATCTGCGCTGCCCCTACCACTCCTGGACCTACGGATTGGACGGCACACTGATCGCCGCCCCCAATATCGCGGAACT<br>GACCGACACCGACGGAGCGAGCATCGACCGGCACCGCTATGGCCTGGTCGCGGTGGCATTGCGGGAGTGGCTGGGCTATGCGTGGGTCTGC<br>CTGGCCGAGGATCCGCCGTCGTTCGAAGACGACGTCGTCGGGTCGGTCACGGCGCGACTCGGCGACGTGAGCGCCATAGACACCTACCGGA<br>TCGAGGCGTTGCAGGTCGGGCGGCGTGTCAGCTATGACGTCGCGGCGAACTGGAAGTTGATCGTCGAGAACTTCATGGAGTGCTATCACTGC

	GCCACCATCCACCCCGAGCTGACCAGAGTGATACCGGAATTCGCACGCGGCCAGGCGGCTCAGCGATCGGTGGGACGCGGCGCCGAATTCG
	GCTCGGCGGTAGCAGGTTTCACCGTGGACGGCCGGGCAGGCTTCACCGCCCTACCCGGAATCAGGCCGGAGCAGGACCGACGCTACTTCGC
	CATCACCGTCAAACCGACGGTGTTCATCAACCTGGTCCCCGACCACGCGATCATCCACCGGATGTTCCCGATCGCCGCCGACCGAACCATCG
	TGGAATGTGACTGGCTCTATGCCCCCGAGGTGGCCGCGGCCGGTGACATTGCGCACTCGGTCGAACTGTTCCATCGCGTCAACGAGCAGGA
	CTTCGACGCCTGCCAGCGGACCCAGCCCGCGATGTCGTCACGCGCCTACCGCACCGGCGGTGTGCTGGTTCCCGCCGAGCACCACATCGCC
	GAATTCCATCAGTGGGTCATCGCACGCATCGGCACCCCCGCGGTGACGGGGTAA

6

Supplementary Table S3. Accuracy of the identification model for all 8,095 strains downloaded from NCBI GenBank

Mycobacterium species	Accuracy
M. tuberculosis	6451/6533 (98.7%)
M. abscessus subsp. *abscessus*	865/869 (99.5%)
M. abscessus subsp. *massiliense*	359/360 (99.7%)
M. avium	208/210 (99.0%)
M. intracellulare	23/23 (100%)
M. kansasii	41/42 (97.6%)
M. fortuitum	24/24 (100%)
M. xenopi	6/6 (100%)
M. scrofulaceum	3/3 (100%)
M. marinum	25/25 (100%)

Supplementary Table S4. Primers used in the multiplex PCR assay to target resistance genotypes

Targeted resistance genotypes	Primer sequences (forward and reverse)
full length *erm*(41) T28	5’ CATATTCATGATGGTGCTGCGT 3’<br>5’ GTATCAGTGCGCTGGTGACTTG 3’
rrl 2270C/G, 2271 C/G	5’ GGCGGTGGTAACTATAACCATCCT 3’<br>5’ ACAGTCTCCCACCTATCCTACACA 3’

Supplementary Figure S1

The plasmid vector pUC57 has a length of 2,710 bp. The plasmid map is as follows: the XbaI/HindIII insertion site and 200 inserted fragments that include the 150 bp amplicon, and 25 bp upstream and downstream sequences.

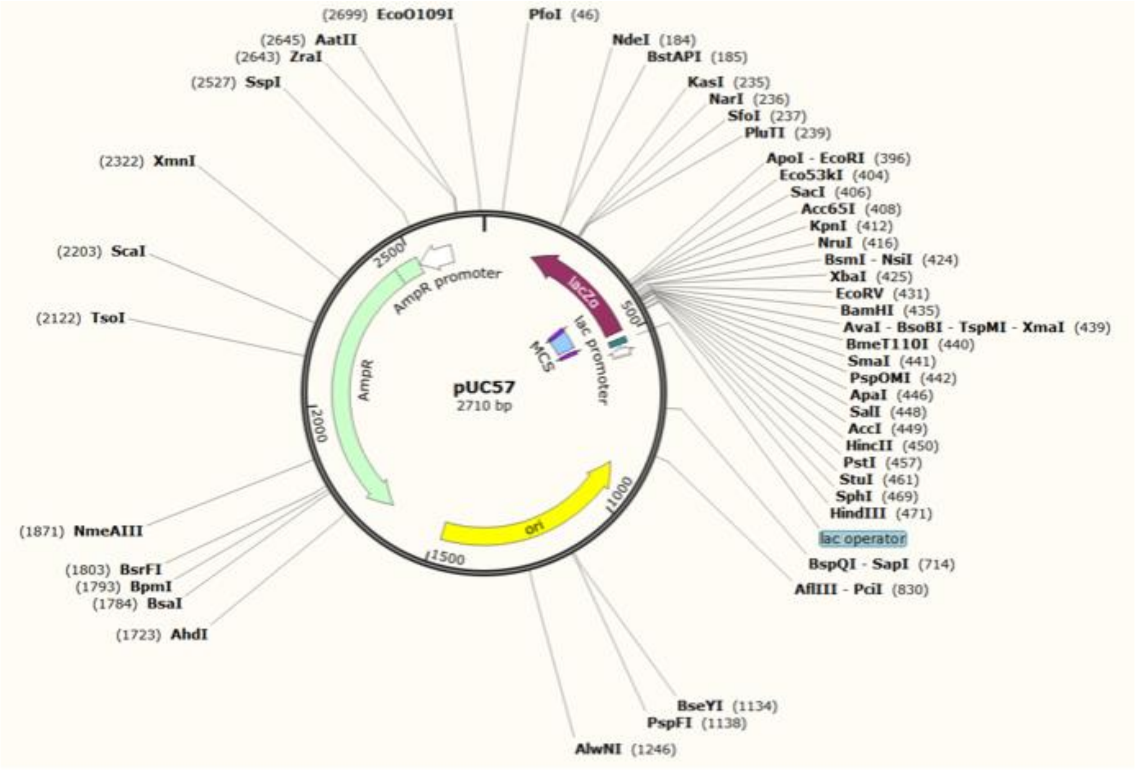

Supplement: Supplemental file 1 [file JCM.00584-20-s0001.pdf]
